# The Impact of Pre- and Probiotic Product Combinations on Ex vivo Growth of Avian Pathogenic *Escherichia coli* and *Salmonella* Enteritidis

**DOI:** 10.3390/microorganisms10010121

**Published:** 2022-01-07

**Authors:** Laura Fuhrmann, Wilfried Vahjen, Jürgen Zentek, Ronald Günther, Eva-Maria Saliu

**Affiliations:** 1 Institute of Animal Nutrition, Department of Veterinary Medicine, Freie Universität Berlin, 14195 Berlin, Germany; wilfried.vahjen@fu-berlin.de (W.V.); juergen.zentek@fu-berlin.de (J.Z.); eva-maria.saliu@fu-berlin.de (E.-M.S.); 2 Fachtierärztliche Praxis für Wirtschaftsgeflügel und Beratung, 39104 Magdeburg, Germany; dr.ronald.guenther@gmail.com

**Keywords:** chicken, inulin, FOS, *Enterococcus faecium*, *Bacillus coagulans*, direct fed microbials, DFM

## Abstract

Due to the global spread of antibiotic resistance, there is a strong demand to replace antimicrobial growth promotors in livestock. To identify suitable additives that inhibit the growth of avian pathogenic *Escherichia coli* O1/O18 and *Salmonella enterica* serotype Enteritidis strains, an ex vivo screening was performed. Inulin and fructooligosaccharides (FOS) were investigated as prebiotics. *Enterococcus faecium* and *Bacillus coagulans* served as probiotic strains. Firstly, the pathogen was anaerobically incubated in caecal digesta from different broiler breeder flocks with the addition of feed additives. Secondly, subsamples of these suspensions were incubated in an antibiotic medium for selective growth of the pathogen. During this step, turbidity was recorded, and lag times were calculated for each pathogen as readout of growth inhibition. Combinations of *E. faecium* with inulin or FOS significantly extended the lag time for *E. coli* compared to control. Moreover, older age was a significant factor to enhance this inhibitory effect. In contrast, the combination of FOS and *B. coagulans* showed shorter lag times for *S*. Enteritidis. Our results indicate that the *E. faecium* strain with prebiotics may inhibit the pathogen proliferation in the studied poultry flocks. Furthermore, our results suggest that prophylactic treatments should be assigned by feed additive, age and animal origin.

## 1. Introduction

The intensification of agriculture has led to a steady increase in poultry meat production over the last few years [1]. This increase in poultry production comes with challenges, as larger holding sizes can pose a risk for infections with pathogenic bacteria such as *Salmonella* [2]. In the EU, *Salmonella* is the most frequently reported foodborne pathogen [3] and associated salmonellosis is the second most reported human zoonotic disease after campylobacteriosis, commonly caused by handling and consumption of poultry products [4]. Among *Salmonella* serovars, *Salmonella enterica* serotype Enteritidis (*S.* Enteritidis) had the highest prevalence in broiler breeding flocks and flock prevalence of *S*. Enteritidis and *Salmonella* Typhimurium (*S.* Typhimurium) were highest in broilers in the EU in 2019 [4]. In addition, *S.* Enteritidis was also the most commonly reported *Salmonella* serovar causing human salmonellosis in the EU in 2017–2019 [4]. In poultry, infections with *S.* Enteritidis usually cause disease in young birds. In adult chickens, infections rarely cause clinical symptoms, but *S*. Enteritidis strains can be found in intact eggs from systemically infected hens, resulting in vertical transmission of the pathogen to offspring [5]. Transmission via eggs can lead to high embryonic mortality and death of newly hatched chicks [5]. In addition to the clinical impact, *Salmonella* also poses a particular health threat by carrying antibiotic resistant genes. In order to control bacterial infections, antibiotics are commonly applied. In turn, this can lead to an increased occurrence of antimicrobial resistance in livestock [6]. Among livestock, poultry shows the highest prevalence of extended-spectrum β-lactamase (ESBL)-producing *Enterobacteriaceae* and thereby represents a reservoir for antibiotic-resistant bacteria of zoonotic relevance [7]. *Escherichia coli (E. coli)* bacteria occur as commensals in the digestive tract of chickens. However, there are also some virulent, avian pathogenic *E. coli* (APEC) strains, which may cause various local or systemic diseases, grouped under the term colibacillosis. In this context, colibacillosis is known as the most common bacterial disease of poultry causing high economic losses [8]. The economic impact may result from lower hatching and production rates, higher mortality, carcass condemnation rates and treatment costs [8,9]. Meanwhile, antibiotic resistant bacteria in livestock pose a global hazard to health, which has led to the ban of antimicrobial growth promoters (AGP) in the European Union [10]. Additionally, both prophylactic and metaphylactic use of antibiotics will be further restricted starting in 2022 [11]. Hence, the demand for effective and safe measures to reduce the spread of antibiotic resistant microbials and to replace AGP is growing worldwide [12]. Research indicates that prebiotics and probiotics can have reductive effects on the gastrointestinal pathogen load [13]. Moreover, probiotic products can reduce the intestinal colonization and transmission of ESBL-producing *E. coli* in poultry [14]. Referring to FAO/WHO and the International Scientific Association for Probiotics and Prebiotics (ISAPP), probiotics are defined as “live strains of strictly selected microorganisms which, when administered in adequate amounts, confer a health benefit on the host” [15]. However, for many probiotics, inconsistent effects are observed [16,17]. In 2016, the ISAPP also stated a definition of the term prebiotic, which reads “a substrate that is selectively utilized by host microorganisms conferring a health benefit” [18]. Similar to probiotics, health benefits provided by prebiotics are inconsistently observed with different responses in individual experimental settings [16,17]. In addition to the different strains and dosages applied in the studies, these varying treatment outcomes could also be due to uneven microbial compositions in the treated animals, which are influenced by host and farm specific environmental factors such as infections [19], feed composition, age, breed and housing conditions [20]. Before administration of feed supplements, these multiple external factors affecting the microbiome need to be addressed. Consequently, a pre-selection of individual pre-and probiotic products adjusted to customized microbial conditions and thus considering host and farm-specific environmental influences, seems to be crucial. Ex vivo assays with digesta content can serve as a suitable preselection method for feed additives to accomplish this approach [21]. Accordingly, it was the aim of this screening study to identify beneficial prebiotics and/or probiotics sufficient to inhibit the growth of an avian pathogenic *E. coli* serotype O1/O18 and *S*. Enteritidis strain under the influence of the chicken caecal microbiome.

## 2. Materials and Methods

### 2.1. Experimental Design

A two-step ex vivo assay was applied according to Zeilinger et al. [21], with minor modifications. Briefly, caecum contents from three broiler breeder flocks were incubated with eight different treatments of pre- and probiotics and a bacterial pathogen in an anaerobic chamber. After 24 h, the supernatant was transferred to an antibiotic medium, allowing only the growth of the respective pathogen. During this second 24 h-incubation, the turbidity (OD_690_) was recorded and, subsequently, OD-data were used to calculate growth parameters for each pathogen as influenced by different treatments. Finally, the lag time variation served as the key indicator for growth inhibition of the pathogenic strains.

### 2.2. Sample Collection

Caecal content was collected from broiler breeders of breeding line Ross 308 (flock A, C) and Cobb 500 (flock B). The breeders were reared on commercial farms in Germany during May 2019 to February 2020. Each flock was kept at a different laying farm, but all farms had the same management and basic structural conditions. Sampling of caeca occurred during routine necropsies (week 25 and week 50) based on an internal health monitoring scheme directly after stunning and killing, which was carried out according to council regulation (EC) No. 1099/2009. The samples were therefore not obtained as part of an animal trial. Caeca of five animals per flock were obtained at each sampling date. Thereafter, the caeca were immediately stored on cardboard in a zipper bag at −18 °C and transported to a laboratory facility. Caecum contents were extracted from the caeca and each 200 mg sample were stored at −80 °C until further use.

### 2.3. Pathogens and Media

Field isolates of an avian pathogenic *E. coli* (serotype O1/O18) and *S.* Enteritidis strains were used to determine growth inhibition via chosen pre- and probiotics. The *E. coli* strain had been isolated from a broiler breeder flock in Germany and was characterized as avian pathogenic by the following virulence associated genes: astA-, iss+, irp2+, papC-, iucD+, tsh-, vat-, cvi/cva+. The *S*. Enteritidis strain was isolated from laying hens.

Both pathogenic strains were stored as cryo stocks (Roti^®^Store cryo vials, Carl Roth GmbH & Co. KG, Karlsruhe, Germany) and cultured in brain heart infusion broth (BHI, Carl Roth GmbH + Co. KG, Germany) throughout the study. Phosphate buffered saline (PBS, pH 7.0, Sigma-Aldrich Chemie GmbH, Taufkirchen, Germany) was used to dilute prebiotics, probiotics and caecum content. In order to reduce oxygen, PBS was supplemented with 0.5 mg/mL L-cysteine and 0.2% resazurin.

Antibiotic resistance patterns of the pathogenic strains were determined with an agar diffusion test on Mueller Hinton Agar II ( Supplementary Table S1).

### 2.4. Determination of Suitable Antibiotic Combinations

Minimal inhibitory concentration (MIC) of antibiotics, against which the pathogens proved to be resistant (trimethoprim, vancomycin, clindamycin, metronidazole, lincomycin), were determined via the broth micro-dilution method in 96-well microplates. To ensure that the antibiotics and their concentrations are suitable to suppress the growth of microbiota in caecum but not the selected pathogen, pool samples for each flock of early laying phase (week 25) and final phase (week 50) were used to verify total growth inhibition of indigenous bacteria at the chosen antibiotic combinations and concentrations. Finally, a combination of clindamycin and lincomycin at a final concentration of 250 µg/mL each proved most suitable for both pathogens in the ex vivo assay.

### 2.5. Pre- and Probiotic Products

Two commercially available prebiotic products were used in this study. Inulin derived from chicory (90%; DP 2-60; Orafti^®^GR, Beneo GmbH, Oreye, Belgium) and fructooligosaccharides (FOS) produced by partial enzymatic hydrolysis from chicory inulin (93–97% β-(2,1)-linkage oligofructose; DP 2-8; Orafti^®^P95, Beneo GmbH, Oreye, Belgium) were diluted in PBS to a concentration of 20 mg/mL and sterile-filtered (0.2 µm cellulose acetate; VWR international, Radnor, PA, USA). *Enterococcus faecium* DSM 7134 (10^10^ cfu/g, Bonvital Pellets, Lactosan GmbH & Co.KG, Kapfenberg, Germany) and *Bacillus coagulans* DSM 32016 (2.5 × 10^9^ cfu/g, TechnoSpore^®^, Biochem, Lohne, Germany) served as probiotic products. Both probiotics are licensed in the EU as gut flora stabilizers in chickens according to council regulation (EC) No. 1831/2003. The final concentration on microplates was 2 mg/mL for prebiotic products and 10^7^ cfu/mL for probiotic products. The final antibiotic mixture (see Section 2.4) was also tested on the probiotic products to guarantee the absence of growth of probiotic bacteria in the assay.

### 2.6. Ex vivo Assay and Determination of Growth Parameters

For the ex vivo assay the pathogenic bacteria were cultivated overnight in 10 mL BHI aerobically at 37 °C. After the preculture, a suspension of 10^5^ cells/mL (*E. coli* strain) or 10^6^ cells/mL (*S.* Enteritidis) was prepared in BHI medium. Each of the following steps were carried out under anaerobic conditions. Prebiotic stock solutions (20 mg/mL) were supplemented with 1 µL/mL of L-cysteine. Dry weighed probiotic products were directly diluted to the final dilution (10^8^ cfu/mL) in PBS. Caecal contents were diluted with PBS (1:10 *v*:*v*) and sedimented for 5 min. Thereafter, the microtiter plates were prepared with 160 µL caecal slurry supernatant, 20 µL pre- or probiotic suspension and/or 20 µL of the pathogenic strain. This resulted in a final concentration of 10^7^ cfu probiotics/mL and 2 mg prebiotics/mL. Each of the two pre- and probiotic products were applied singly (20 µL PBS added instead of other component) or in combination, resulting in eight different treatments. Respective negative- and positive controls were investigated simultaneously. The microtiter plate was then incubated anaerobically at 37 °C for 24 h.

In the second step of the assay, 15 µL of the incubated caecal samples were inoculated into BHI medium supplemented with clindamycin and lincomycin (final conc. 250 µg/mL each) and again incubated anaerobically by airtight sealing the microtiter plate with a transparent gastight film (ViewSeal^TM^, Greiner Bio-One GmbH, Leipzig, Germany). Subsequently, the 24 h-incubation was carried out in a microplate reader (Tecan Infinite200Pro, Männedorf, Switzerland) at 37 °C. Turbidity was recorded every 5 min with lateral shaking for 10 s prior to each measurement. Optical density data were used to calculate growth parameters (lag time, specific growth, maximum OD) by a 3-parameter sigmoidal equation for exponential growth using SigmaPlot version 11.0 (Systat Software Inc., Chicago, IL, USA). As described in previous studies, lag time was selected as the most appropriate growth parameter to determine bacterial fitness [21,22]. Pathogenic strains, that showed no growth within 24 h after the first incubation were set to a lag time of 24 h, to include non-growth after treatment in the analysis. Overall, the complete data set consists of 270 assays per pathogenic strain, derived from three different farms at two sampling time points, with nine different treatments tested, including positive control.

### 2.7. 16S rDNA Sequencing

DNA was extracted from caecal samples (100 mg) of each individual animal included in the ex vivo assay. A commercial kit (QIAamp PowerFecal Pro DNA Kit, Qiagen, Hilden, Germany) was applied according to the manufacturer’s instructions. Before the beat–beating process, an additional lysis step at 65 °C for 10 min was integrated into the protocol. Finally, obtained DNA extracts were sequenced on an Illumina NextSeq500 sequencer (LGC, Berlin, Germany) with 150 bp-paired reads using 16S rDNA primers 341f and 785r. Demultiplexing was performed using Illumina bcl2fastq (v. 2.17.1.14) and a combination of paired-end reads was performed using BBMerge (v. 34.48). Subsequently, the resulting 16S rDNA sequences were analyzed using the QIIME2 pipeline [23] and the SILVA SSU database [24]. Quality control and sequence counts were determined using DADA2 [25]. Sequence variants with fewer than five counts were excluded from further analysis in order to reduce bias from potential sequencing errors and increase confidence of sequence reads [26]. Sequence reads were normalized by rarefaction with an equal representation of 10,000 sequences per sample [27].

### 2.8. Statistical Analysis

Lag time values are presented as mean values for each farm, treatment and pathogenic strain. Subtractive lag time was calculated in order to present the effect of feed additives to delay (positive values) or accelerate (negative values) the pathogenic growth. Therefore, the lag times of the positive controls were subtracted from the treatment groups. Statistical analyses were performed with the software SPSS 25 (IBM SPSS 25; Armonk, NY, USA). Initially, boxplot diagrams were drawn up and optically detected extreme outliers (more than three interquartile ranges below or above 25 or 75 quartile) were excluded for each treatment group from the following analyses. Statistical analyses were calculated using ANOVA and post hoc Tukey-HSD for normally distributed data, whereas the non-parametric Kruskal–Wallis test with Bonferroni correction followed by the Mann–Whitney test was applied for non-normally distributed data. Differences were considered as significant at *p* ≤ 0.05 and *p* = 0.05–0.10 were considered trends. The online tool ClustVis (https://biit.cs.ut.ee/clustvis/, accessed on 19 November 2021) was used to generate principal component analysis analyses of the 16S rDNA sequencing data [28].

## 3. Results

### 3.1. Original Bacterial Composition of Flock Caecal Contents

**Figure 1:**
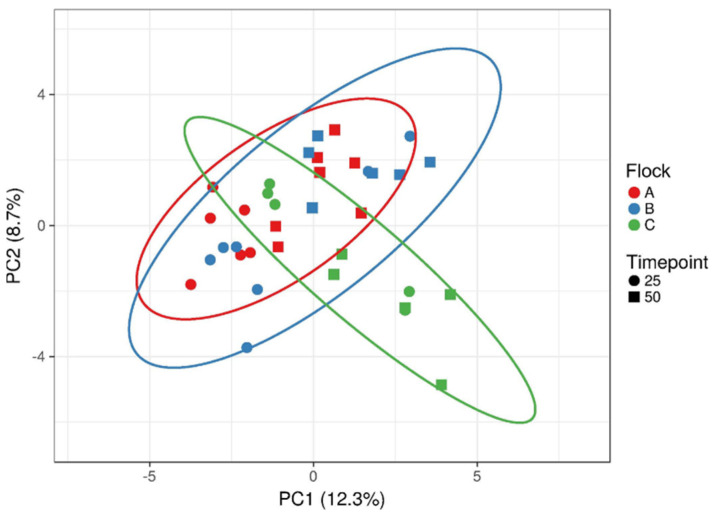
Principal component analysis on the whole 16S rDNA data set visualizing differences between flocks (A, B, C) and animal age (25 and 50 weeks of age). Different flocks are indicated by different colors, while timepoint 25 is displayed as circle and timepoint 50 as square. The prediction eclipses show a *p* = 0.95 probability for new observations.

16S rDNA sequence data was used to inspect the caecum samples for possible differences in bacterial composition. Supplementary Tables S2 and S3 show the comparison of the dominant (>1% total) bacterial genera of the three flocks during the early or late laying phase. Age had less influence on the dominating *Bacteroides*, *Lactobacillus* and an unknown *Lachnospiraceae* genus, but the abundance of the unknown *Lachnospiraceae* increased in 50-week-old animals at the expense of *Bacteroides* and *Lactobacillus*. However, flock specific differences at both ages were observed for a range of bacterial genera. Thus, compared to other flocks, flock B showed decreased abundance of the genus *Ruminococcaceae* UCG-015 or *Collinsella* in the early or late laying phase, respectively. Of note was also flock C, which showed a drastic decrease in abundance for *Bacteroides* and *Blautia*, as they were replaced by an increased abundance of a *Christenellaceae* R-7 group genus in samples from 50-week-old chicken. Furthermore, flock C seemed to show a different bacterial composition than the other flocks. We therefore used a principal component analysis on the whole 16S rDNA data set to visualize microbiota differences between flocks and animal age (Figure 1). As observed for the dominant genera, few differences between 25 and 50 weeks of age were noted. However, overall, flock C displayed a different cluster formation than flocks A and B. This was also evidenced by a shift in diversity indices for this flock at 50 weeks of age (Table 1).

### 3.2. Ex Vivo Assay

**Figure 2:**
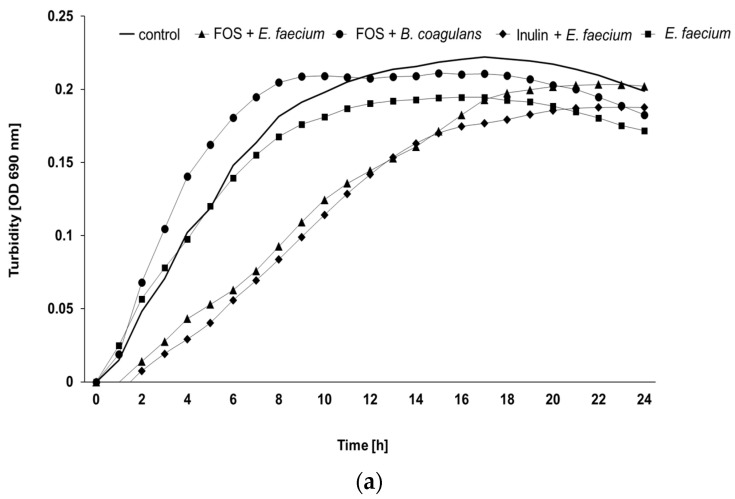

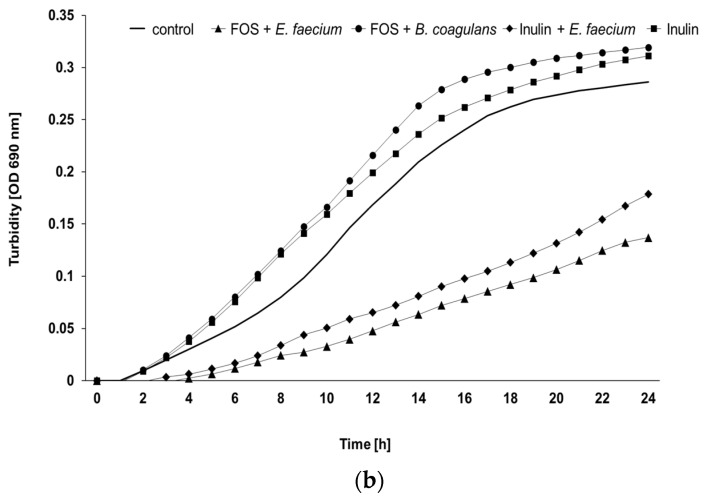
Growth curves presenting the fitness of the pathogenic strains after incubation with different additives in caecal samples from: (**a**) 25 weeks old broiler breeders (*E. coli*) and (**b**) 50 weeks old broiler breeders (*S.* Enteritidis) (mean values, *n* = 15, all farms included FOS = fructooligosaccharides).

The antibiotic combination of clindamycin and lincomycin at 250 µg/mL was sufficient to inhibit the growth of the caecal microbiota within the non-inoculated cultures of the broiler breeder samples. This was the prerequisite to specifically monitor the proliferation of the pathogenic strains after incubation with the caecal matrix and the additives. Pathogens inoculated in caecal slurries consistently showed growth in all positive controls. In general, the *S.* Enteritidis strain displayed longer lag times than the *E. coli* strain (Figure S1). The different treatments modified the growth of the pathogens in distinct ways. Typical growth curves of *E. coli* O1/O18 and *S.* Enteritidis displaying the effects of certain additives are shown in Figure 2. Compared to positive controls, the combinations of the prebiotics (inulin or FOS) with the probiotic product *E. faecium* extended the lag times for both *E. coli* and *S.* Enteritidis, by decreasing their fitness after incubation. Other supplements such as inulin or the combination of FOS and the *Bacillus* probiotic shortened the lag time in comparison to the control and therefore seemed to support the pathogen fitness.

Lag time values for the *E. coli* and *S.* Enteritidis strains are shown in Table 2 and Table 3, respectively. Longer lag times compared to the control indicate poorer fitness of the respective pathogen, while shorter lag times characterize a better fitness after the first incubation.

### 3.3. Ex Vivo Growth of E. coli O1/O18 after Incubation with the Additives

Lag time data of the *E. coli* strain are presented in Table 2. Significant differences between treatments were observed in all flocks of the final laying phase (50 weeks), but at week 25, differences were only observed for flock A. Older age led to significantly prolonged lag times for the single addition of probiotics, FOS and combinations of *E. faecium* with the investigated prebiotics. Additionally, latter combinations displayed a significant extension of at least eight hours compared to positive control in the overall analysis (*n* = 30). Independently, the *Bacillus* probiotic and the prebiotic FOS increased the lag time of *E. coli* numerically, however when combined, shorter lag times were observed in the general analysis. A slight numerically improved fitness of the pathogenic *E. coli* strain was also found after incubation in caecal slurries supplemented with inulin alone or in combination with *B. coagulans*. This is in contrast to the decreased fitness observed for combinations of the *E. faecium* probiotic with the prebiotic’s inulin and FOS. The caecal slurries supplemented with the *E. faecium*-prebiotic combinations frequently revealed longer lag times compared to positive controls in all flocks. In flock A the combination of FOS and *E. faecium* significantly extended the lag time towards positive control in the early laying phase, while flock B and C revealed a significant increase in lag time values only in the final phase (50 weeks). Moreover, in flock B and C, the addition of the *Enterococcus* probiotic with inulin also led to significantly lower fitness of the pathogen at 50 weeks of age. Samples of flock B showed the most direct effects of *Enterococcus* and prebiotics combinations, as no growth was observed in samples of the final laying phase. Numerically, the prebiotic FOS alone also reduced the fitness of the pathogen at 50 weeks of age. In contrast, the combination of FOS and the probiotic *B. coagulans* more or less showed numerically shorter lag times towards positive control across all flocks and ages. The combination of inulin and *B. coagulans* displayed the same effects in the flocks of the final laying phase. Inconsistent effects were observed for all other supplements (inulin and probiotics alone) across the different flocks.

**Figure 3:**
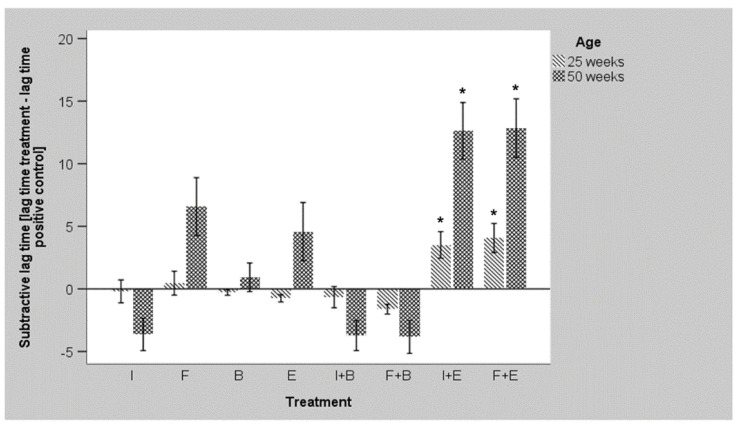
Comparison of different treatments and ages regarding the lag time [h] of *E. coli* O1/O18 (I = Inulin, F = FOS, E = *Enterococcus faecium*, B = *Bacillus coagulans*, + = combination; mean values, *n* = 15 for each age, all farms included, error bars indicate the SEM, significant differences compared to positive control are indicated by asterisks).

Figure 3 depicts a summary of the flock data for the *E. coli* strain and highlights the differences between age groups in relation to each treatment. Negative subtractive lag time symbolizes better pathogen growth than in positive controls, whereas positive values indicate a reduced pathogen fitness. In both week 25 and week 50, the combination of inulin or FOS with the probiotic *E. faecium* led to the strongest inhibition of pathogen growth. Compared to positive control significant differences were only observed for these combinations with accented effects at later age. Numerically, the single application of FOS and *E. faecium* also impaired pathogen fitness, whereas inulin and combinations of prebiotics with *B. coagulans* increased pathogen survival in samples of 50 weeks.

### 3.4. Ex Vivo Growth of S. Enteritidis after Incubation with the Additives

The impact of pro- and prebiotic addition to the *S.* Enteritidis strain in caecal content of broiler breeders is depicted in Table 3. Similar to *E. coli*, significant differences between the treatments were observed in all flocks at 50 weeks and, additionally, in flock A in the early laying phase. Contrary to the *E. coli* strain, age had a significant impact on fitness of the *Salmonella* strain in each treatment, with prolonged lag times in the final laying phase. The overall analysis showed the highest lag times of the *Salmonella* strain in caecal slurries incubated with *E. faecium* and inulin or FOS. On average, these treatments resulted in numerically delayed growth by nearly 4–5 h compared to positive controls. The treatment with a combination of FOS and the *B. coagulans* probiotic product tended to improve the fitness of the pathogenic strain instead. Moreover, the *E. faecium*-prebiotic combinations revealed significantly higher lag times for *S.* Enteritidis compared to the single application of prebiotics or their combinations with the *Bacillus* strain in the general analysis. However, the pathogenic *Salmonella* strain showed no growth inhibition (flock A, C) or low lag time reduction of three hours (flock B) in samples from the early laying phase with the *E. faecium*-prebiotic treatments. When prebiotics were incubated together with the *Bacillus* probiotic, reductions in lag time were seen across all flocks and ages. Furthermore, the combination of the *Bacillus* probiotic with FOS led to significantly shorter lag times compared to positive control and single addition of the probiotic strain for incubations with early laying phase samples. Similar to the combination with *Bacillus*, the single application of inulin also revealed shorter lag times in each age group. This effect was shown to be statistically significant in flock A at 25 weeks. Moreover, a single application of prebiotics led to significantly lower lag times compared to their combinations with *E. faecium,* not only in the overall analysis but also in the final laying phase. Although this extension in lag time was not significant compared to positive controls, data show a rather drastic prolongation of 6–8 h at 50 weeks. As with the *E. coli* strain, flock B showed numerically the clearest effects of the *Enterococcus*-prebiotic combinations with no detectable growth during 24 h in this age group.

**Figure 4:**
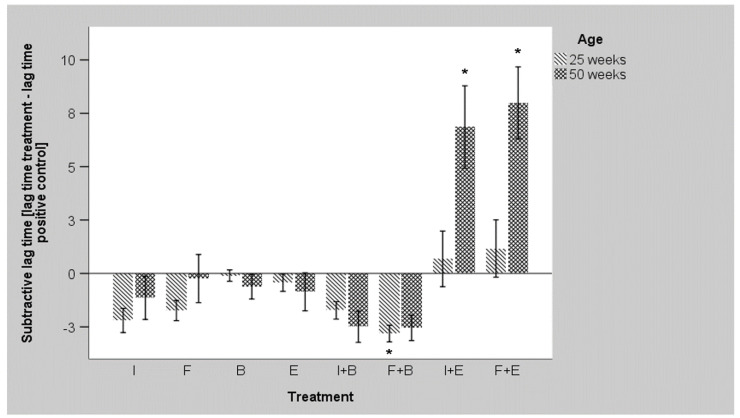
Comparison of different treatments and ages regarding the lag time [h] of *S.* Enteritidis (I = Inulin, F = FOS, E = *Enterococcus faecium*, B = *Bacillus coagulans*, + = combination; mean values, *n* = 15 for each age, all farms included, error bars indicate the SEM, significant differences compared to positive control are indicated by asterisks).

Figure 4 summarizes the individual flock data for the *S.* Enteritidis strain and shows the differences between age groups in relation to each treatment. Only the combination of the probiotic *E. faecium* with inulin or FOS seemed to reduce fitness of the pathogen. Compared to positive controls, significant differences were observed for both combinations at week 50. In contrast, the combination of the *Bacillus* probiotic with FOS significantly increased the fitness in samples of week 25.

## 4. Discussion

An ex vivo assay was employed to investigate the inhibitory effects of four pre- and probiotic products and their combinations on the growth of a pathogenic *E. coli* and *S.* Enteritidis strain in a complex caecal matrix. In chicken, when aiming to modulate the microbiota, the crop and caecum are of high interest. Both compartments show a relatively low digesta passage time compared to the small intestines and, hence, potentials to impact on performance and health via microbiota are revealed. The crop microbiome can be modified by feed additives, which may be the first challenge for an ingested pathogen and, hence, may interrupt the infection chain at an early stage [29]. In fact, this type of assay has been used to monitor the survival of an antibiotic resistant *E. coli* in broiler chicken [30]. However, the highest concentration of bacteria is commonly observed in caecal contents and, thus, this compartment is of utter importance for animal health and performance, but also for the transmission of pathogens via faeces. This was the motivation for choosing a caecal matrix for the assay. When investigating the impact of feed additives on the hindgut microbiota, their fate in the small intestine must be considered. The easily fermentable FOS can reach the large intestine without being degraded by digestive enzymes in the small intestine [31]. Thereby, FOS and its polymers hold a potential to modulate the hind gut microbiota and fermentation profile in vivo [32,33,34]. Similarly, in vivo effects in the large intestine have also been proven for the probiotic species used in our study [35,36].

Regarding the final antibiotic mixture for our ex vivo assay, it was challenging to find a combination that would inhibit the growth of the gut microbiota but at the same time allow the growth of the investigated pathogens. This was especially true for young broilers and led to the exclusion of these animals from the assay due to project time constraints. Even considerably high concentrations (250 µg/mL) of five potent antibiotics (clindamycin, vancomycin, metronidazole, trimethoprim and lincomycin) were not sufficient to completely inhibit background growth in preliminary studies (data not shown). This may serve as a reminder that a range of antibiotic resistant bacteria occur in the intestinal tract of healthy broilers that are not found in broiler breeders [37]. Moreover, this also mirrors the hazard of antibiotic resistant microbials arising from poultry products in the field [7]. Subsequently, considering the vertical transmission pathways of pathogens to broilers, the current study focused on the broiler parent stocks.

A limitation of the current experimental design was the small sample size, as not all strong numerical effects turned into mathematically relevant significant differences. Thus, to identify farm-specific beneficial additive combinations, higher sample sizes per single farm would allow higher replicate numbers with higher mathematical confidence.

Finally, the method investigates short-term effects of the supplements against certain pathogenic bacteria without interference of the host. Therefore, the ex vivo screening is insufficient to draw conclusions about the benefits of feed additives on the host. Prebiotics and probiotics have different modes of action and in some cases interact with the immune system and other host related factors rather than directly on gut bacterial populations [38,39]. Subsequently, it is not possible to speculate about the health-promoting properties of individual pre- and probiotics with this method. Nevertheless, firstly, this assay was able to elucidate certain pre- and probiotic combinations that showed even an increased fitness of the pathogen in question. Secondly, this screening method provided promising insights into farm-, age-, and pathogen-dependent effects of feed additives on the intestinal microbiota. It can therefore serve as preselection method to avoid expensive and unprofitable applications of supplements, especially in the context of individual solutions for specific farms. It is also important as a step regarding animal welfare as it serves as a pre-selective assay which helps to reduce the animal numbers needed for in vivo trials. Thirdly, due to the microtiter plate format of the assay, a large number of feed additives can be tested simultaneously. All in all, the method represents a reasonable compromise to draw first conclusions on the effect of feed additives on the intestinal microbiota compared to cost-intensive animal experiments and limited in vitro studies [21].

As the intestinal tract of poultry can be seen as a reservoir for APECs [40], one approach to control *E. coli* related diseases is to suppress their proliferation in the intestine. Interestingly, only the combination of the *Enterococcus* probiotic with either prebiotic product had a significant impact on the fitness of the *E. coli* strain at both week 25 and 50. Furthermore, the routes of transmission of the zoonotic *Salmonella* are varied and both vertical and horizontal transmission are possible [5]. Hence, effective control strategies such as feed additives to replace AGP are urgently needed throughout the poultry production chain. In addition, preventing *S*. Enteritidis from caecal colonization by the use of feed additives might be a promising approach, as this species has a high potential to colonize the poultry hind gut compared to other *Salmonella enterica* serotypes [41]. Reducing concentrations in the caecum may also reduce the risk of transmission between animals and the hazard of product contamination. Similar to results for *E. coli*, only a few feed additive combinations showed a significant impact on the fitness of the *S*. Enteritidis strain. Again, the *E. faecium* probiotic in combination with either prebiotic product proved to be the most effective treatment to reduce Salmonella fitness, but only in older animals. In previous in vivo studies desirable symbiotic effects of *E. faecium* with FOS have also been described [42,43]. *E. faecium* is a Gram-positive, facultative anaerobic bacterium, indigenous to the digestive tract of chicken [44]. Hexose fermentation yields L-lactate as its main metabolite [45] and the ability of *E. faecium* to grow in the presence of FOS has been described previously [46]. As pH changes and bacterial metabolites were not measured within this study, it can only be hypothesized which molecular mechanisms caused the observed lag time delays. It is tempting to correlate probiotic lactate production of *E. faecium* to the decreased pathogen fitness, as lactate production frequently results in lower pH. In a previous in vitro study *E. faecium* cultures showed a pH decline from 6.7 to 5.0 within 10 h in FOS-supplemented media [31]. Furthermore, a decreased pH can pose an inhibitory effect on several pathogens [47]. This might be due to a supportive effect on antimicrobial compounds secreted by lactic acid bacteria [48] and/or the cytotoxic effect of lipophilic acids and lowered pH itself [49]. However, the quantitative comparison of 10^5^ probiotic cells/mL slurry against a background of approximately 10^11^ indigenous cells/mL (with about 10^9^ indigenous lactobacilli/mL) shows that lactate production and the consecutive reduction in pH by probiotic strains cannot be a relevant factor. The species *E. faecium* has the ability to produce bacteriocins, which may work directly against a multiplication of pathogens [50]. Although it is not known whether the *E. faecium* strain used in this study possesses any bacteriocins or not, it may explain the numerical decrease in pathogen fitness in the late laying phase. Another possible explanation might be the stimulative effect of the probiotic strain on selected indigenous bacteria and thereby, a modulation of the sample matrix. Accordingly, the *E. faecium* strain used in our assay has been shown to increase lactobacilli counts in excreta samples in laying hens [51]. Additional enrichment with this group of bacteria may have occurred during the first incubation step. Furthermore, indigenous bacterial metabolite concentrations are inevitably enriched during the incubation in caecal slurries. Hence, the negative influence on the growth of the pathogenic strains by *E. faecium* addition may be of multifactorial origin. It can be speculated that the *E. faecium* probiotic by itself prepared certain bacterial groups for a higher potential to reduce *E. coli* fitness, but that the prebiotics delivered the needed nutrient and energy supply for bacterial metabolism. In fact, it is known from another species of the *Enterococcus* genus that the metabolism of FOS can stimulate the bacterium to produce proteins favourable for a probiotic strain [52]. Thus, these additive combinations could be termed synbiotic.

The results observed for the *Enterococcus* probiotic are in sharp contrast to respective results with the *Bacillus* probiotic product. Here, combinations with both prebiotics even numerically increased the fitness of the *E. coli* strain in both age groups. Despite the inhibitory effect on *S*. Enteritidis in animal trials [53], *B. coagulans* did not impair the fitness of the *Salmonella* pathogen in our study and led to significant better pathogen fitness in combination with FOS in the early laying phase. Obviously, the *Bacillus* probiotic modulated the response of the caecal microbiota to an environment less detrimental to the survival of the pathogenic *E. coli* and *S*. Enteritidis strains. Similar to our findings, another *Bacillus coagulans* strain was not effective to inhibit *S.* Enteritidis growth in an agar inhibition assay [46]. Obviously, for these reasons, *B. coagulans* and its mentioned combinations are not recommended for prevention against the investigated pathogens. However, in vivo effects may differ as the mode of action of this product may be related to interactions with the host, which could not be addressed with this assay. Indeed, it has been shown that *B. coagulans* strongly interacts with the hosts immune system [54]. Previous results from another *B. coagulans* strain did show beneficial effects on the host that were mainly related to body weight gain and immune stimulation [55] and, thus, the outcome in the current screening study with this probiotic does not automatically exclude any positive effects in general. However, strain specific effects may also explain these different outcomes.

In the current study, both single applications of the prebiotics failed to significantly reduce *E. coli* fitness. A possible explanation might be, that they failed to stimulate competitive indigenous microbiota sufficiently. Furthermore, in contrast to the numerical effects of FOS on *E. coli* inhibition, a single application of inulin rather improved pathogen fitness in caecal slurries from 50 weeks old broiler breeders. This could reflect a lower utilization rate of the caecal microbiota for polysaccharides similar to inulin compared to oligosaccharides such as FOS [56]. Another possible explanation might be, that the pathogens were able to utilize this prebiotic product to their own benefits. *S.* Enteritidis can ferment FOS, when other carbon sources such as pure glucose or fructose are available [46]. In our ex vivo assay, glucose was provided by the BHI medium, which together with FOS may have resulted in better growth of this *S.* Enteritidis strain, when exposed to the single prebiotic. This could also serve as an explanation for better pathogen survival with inulin, as inulin is a polymer of FOS. In rats, it has already been shown that both inulin and FOS can cause increased colonization of *S.* Enteritidis in caecal contents [57].

Overall, the feed additives modified the fitness of the pathogenic strains much more frequently in 50-week-old animals. Age is known to be a major factor of microbiome shifts in chickens [20]. From data on bacterial diversity, we infer that a more diverse bacterial composition in caecal contents might enhance the response of the caecal microbiota to a certain pre- or probiotic treatment. Furthermore, flock-specific differences were noted especially for the application of single pre- and probiotics, which might be also due to the different breed [20]. This varied treatment response together with the results of the principal component analysis imply that not all studied flocks acted similar and, therefore, screening studies such as the current may identify farm-specific solutions for the application of feed additives.

Age- and flock specific differences in pathogen inhibition were most likely due to different bacterial compositions in the caecal samples, since feed influence due to identical rations could be largely excluded. In addition, the herds lived at the same time and received their feed from the same feed mill. Unfortunately, an analysis of the samples after incubation was not possible due to prohibitively high sample numbers, but the initial bacterial composition at the start of the experiment is known. A comparison of 16S rDNA data from 25- and 50-week-old animals indeed showed changes in abundance among the most dominant bacteria, as well as differences between individual flocks. It is highly likely that the addition of the pre- and probiotic products changed the bacterial composition and activity, depending on the starting conditions. This suggests that the caecal microbiota responded differently and, thus, observed changes in the fitness of the pathogenic strains may depend in large parts on responsive microbiota. Different microbial starting conditions might induce distinct levels of pathogen fitness after exposure to certain feed additives. Farm-specific differences in microbiome composition have also been described in sows and piglets [58]. This indicates the importance of treatment preselection, not only for poultry but also for other monogastric livestock. Correspondingly, promising results for preselection of prebiotics and probiotics for sows have already been observed in another ex vivo experiments, similar to the present work [21].

Considered together, the growth inhibition of the pathogenic bacteria in our study was most likely due to synergistic effects of the probiotic strain *E. faecium* and the fermentation of the soluble prebiotic products by the indigenous bacteria, while the *Bacillus* probiotic strain was not able to modify the microbiota favourably. Apart from a significant improvement in *Salmonella* fitness in one flock due to inulin, the single application of the prebiotics rarely influenced the fitness of the pathogens.

## 5. Conclusions

Overall, the results of the current study demonstrate the beneficial effect of a probiotic *E. faecium* strain in combination with inulin or FOS to inhibit the survival of an avian pathogenic *E. coli* O1/O18 and a *S.* Enteritidis strain under the influence of the chicken caecal microbiome. Furthermore, older age was a significant factor to enhance the inhibitory effect of the mentioned combinations. Surprisingly, the application of a single prebiotic either showed no significant effects or even increased pathogen fitness. Furthermore, not all flocks responded equally to the application of certain pre- and probiotic combinations, and the effects of feed additives differed among pathogens in some cases. Thus, farm- and pathogen specific solutions may be needed to address farm-specific problems.

## Figures and Tables

**Table 1:** Bacterial diversity indices in caecal samples of broiler breeders in different flocks.

	Broiler Breeder Flock				
Age	A	B	C	SEM	*p*-Value *
25 weeks					
Richness	135.29	173.8	139.17	8.5	0.077
Shannon-Index	4.07	3.61	3.81	0.10	0.161
Evenness	0.833 ^b^	0.705 ^a^	0.775 ^a,b^	0.021	0.007
50 weeks					
Richness	177.29 ^b^	131.5 ^a^	246.0 ^c^	14.0	0.016
Shannon-Index	4.40 ^b^	4.00 ^a^	4.64 ^b^	0.07	0.03
Evenness	0.852 ^b^	0.822 ^a^	0.849 ^b^	0.053	0.046

* = Kruskal–Wallis Test. ^a,b,c^ = different superscripts indicate significant differences within a row (Mann–Whitney Test).

**Table 2:** Impact of pre- and probiotic products on the lag time of a pathogenic *Escherichia coli* O1/O18 strain after incubation in caecal slurries [h].

			Prebiotic Products		Probiotic Products		Bacillus and Prebiotic		Enterococcus and Prebiotic		
Age (Weeks)	Flock	Control	Inulin	FOS	Bacillus	Enterococcus	Inulin	FOS	Inulin	FOS	*p*-Value Treatment
25	A	4.99 ^a,b^	4.90 ^a,b,c^	5.29 ^a,b,c^	4.60 ^a^	4.02 ^a^	4.69 ^a^	3.51 ^a^	9.29 ^b,c^	9.28 ^c^	0.011
	B	5.79	5.00	6.31	4.80	4.34	4.78	3.84	7.99	8.23	0.441
	C	4.28	4.52	4.84	4.78	4.47	4.57	2.84	8.32	9.76	0.137
	All	5.02 ^a,b^	4.81 ^a,b^	5.48 ^a,b^	4.73 ^a,b^	4.27 ^a^	4.68 ^a,b^	3.40 ^a^	8.53 ^b^	9.09 ^b^	<0.001
50	A	6.43 ^a,b^	4.39 ^a,b^	5.36 ^a,b^	9.84 ^b^	6.24 ^a,b^	3.60 ^a,b^	2.38 ^a^	12.48 ^b^	12.46 ^a,b^	0.009
	B	5.95 ^a^	7.73 ^a,b^	17.70 ^a,b^	7.53 ^a,b^	17.13 ^a,b^	5.81 ^a,b^	5.91 ^a,b^	24.00 ^b^	24.00 ^b^	<0.001
	C	7.35 ^a,b^	3.35 ^a,b^	16.36 ^b,c^	5.11 ^a,b^	10.06 ^a,b,c^	3.70 ^a^	5.72 ^a,b^	21.11 ^c^	21.75 ^c^	0.006
	All	6.57 ^a^	4.90 ^a^	13.14 ^a,b^	7.49 ^a,b^	11.15 ^a,b^	4.29 ^a^	4.10 ^a^	19.20 ^b^	19.41 ^b^	<0.001
All	All	5.80 ^a,b^	4.84 ^a,b^	9.31 ^b,c^	6.11 ^a,b^	7.71 ^a,b^	4.51 ^a,b^	3.68 ^a^	13.86 ^c^	14.25 ^c^	<0.001
*p*-value age		0.250	0.726	0.016	0.005	0.015	0.796	0.643	<0.001	0.003	

^a,b,c^ = different superscripts indicate significant differences within a row (Kruskal–Wallis, Bonferroni test at *p* ≤ 0.05), results are presented as means, sample size *n* = 5 per flock and age group, *n* = 15 all per age group, *n* = 30 all per treatment.

**Table 3:** Impact of pre- and probiotic products on the lag time of a pathogenic *Salmonella* Enteritidis strain after incubation in caecal slurries [h].

			Prebiotic Products		Probiotic Products		Bacillus and Prebiotic		Enterococcus and Prebiotic		
Age (Weeks)	Flock	Control	Inulin	FOS	Bacillus	Enterococcus	Inulin	FOS	Inulin	FOS	*p*-Value Treatment
25	A	7.83 ^b^	5.25 ^a^	6.05 ^a,b^	7.78 ^b^	7.94 ^b^	6.47 ^a,b^	6.25 ^a,b^	6.35 ^a,b^	7.68 ^b^	0.043
	B	9.24	7.48	7.36	9.42	9.15	7.20	6.08	12.34	12.92	0.083
	C	9.44	7.19	7.89	9.03	8.09	7.66	5.75	9.81	9.44	0.065
	All	8.84 ^b^	6.64 ^a,b^	7.10 ^a,b^	8.74 ^b^	8.39 ^a,b^	7.11 ^a,b^	6.03 ^a^	9.50 ^a,b^	10.20 ^b^	<0.001
50	A	10.72 ^a,b,c^	8.26 ^a^	9.27 ^a^	11.04 ^a,b,c^	9.63 ^a,b^	8.78 ^a^	8.49 ^a^	16.23 ^b,c^	16.87 ^c^	0.029
	B	13.62 ^a,b^	15.90 ^a,b^	18.25 ^a,b^	11.80 ^a^	13.54 ^a,b^	11.53 ^a^	12.05 ^a^	24.00 ^b^	24.00 ^b^	<0.001
	C	11.74 ^a,b^	8.53 ^a,b^	7.92 ^a^	11.43 ^a,b^	10.33 ^a,b^	8.29 ^a,b^	7.93 ^a^	16.41 ^a,b^	19.21 ^b^	0.011
	All	12.03 ^a,b,c^	10.90 ^a^	11.81 ^a,b^	11.42 ^a,b,c^	11.17 ^a,b^	9.54 ^a^	9.49 ^a^	18.88 ^b,c^	20.02 ^c^	<0.001
All	All	10.43 ^a,b,c^	8.77 ^a^	9.45 ^a,b^	10.08 ^a,b,c^	9.78 ^a,b,c^	8.32 ^a^	7.76 ^a^	14.19 ^b,c^	15.02 ^c^	<0.001
*p*-value age		0.004	0.004	0.010	0.002	0.009	0.005	0.004	<0.001	<0.001	

^a,b,c^ = different superscripts indicate significant differences within a row (Kruskal–Wallis, Bonferroni test at *p* ≤ 0.05), results are presented as means, sample size *n* = 5 per flock and age group, *n* = 15 all per age group, *n* = 30 all per treatment.

## Data Availability

The data presented in this study are available on request from the corresponding author.

## Supplementary Materials

The following supporting information can be downloaded at: https://www.mdpi.com/article/10.3390/microorganisms10010121/s1. Table S1: Resistance profile of the pathogenic strains *Escherichia coli* O1/O18 and *Salmonella* serotype Enteritidis, tested with agar diffusion method. R = resistant, S = sensitive.; Table S2: Relative abundance of the most dominant bacterial genera in caecal samples of 25-week-old broiler breeders in different flocks (*n* = 5).; Table S3: Relative abundance of the most dominant bacterial genera in caecal samples of 50-week-old broiler breeders in different flocks (*n* = 5).; Figure S1: Mean control growth curves of the pathogenic strains *Escherichia coli* O1/O18 (A) and *Salmonella* Enteritidis (B) after incubation in chicken caecal slurries (*n* = 30), error bars indicate the SEM.
