# Supplementary material for: The Impact of Pre- and Probiotic Product Combinations on Ex vivo Growth of Avian Pathogenic Escherichia coli and Salmonella Enteritidis
Source: Microorganisms. 2022 Jan 7;10(1):121. doi: 10.3390/microorganisms10010121 (PMC8778165; doi:10.3390/microorganisms10010121)
Supplement: Supplementary file 1 [file microorganisms-10-00121-s001.zip › microorganisms-1533481-supplementary.pdf]

Supplementary Material

Table S1. Resistance profile of the pathogenic strains *Escherichia coli* O1/O18 and *Salmonella* serotype Enteritidis, tested with agar diffusion method. R = resistant, S = sensitive.

**Antibiotics**	**Concentration**	***E. coli* O1/O18**	***S*. Enteritids**
Amoxicillin	25 µg	R	S
Ampicillin	25 µg	R	S
Bacitracin	10 units	R	R
Cefacetril	30 µg	S	S
Cefalexin	30 µg	S	S
Chloramphenicol	30 µg	S	S
Ciprofloxacin	5 µg	S	S
Clindamycin	10 µg	R	R
Doxycyclin	30 µg	S	S
Erythromycin	30 µg	S	S
Framycetin	100 µg	S	S
Fusidinsäure	10 µg	R	R
Gentamycin	30 µg	S	S
Kanamycin	30 µg	S	S
Lincomycin	15 µg	R	R
Metronidazol	5 µg	R	R
Ofloxacin	5 µg	S	S
Oxacillin	5 µg	R	R
Rifampicin	30 µg	S	S
Streptomycin	25 µg	R	S
Sulfamethoxazol	25 µg	R	R
Sulfamethoxazol/Trim.	25 µg	R	S
Tetracyclin	30 µg	S	S
Trimethoprim	5 µg	R	S
Vancomycin	5 µg	R	R
Tylosin	30 µg	R	R
Marbofloxacin	5 µg	S	S
Piperacillin	75 µg	S	S

Cefotaxime	5 µg	S	S
Amoxicillin	25 µg	R	S
Ampicillin	25 µg	R	R
Bacitracin	10 units	R	S
Cefacetril	30 µg	S	S
Cefalexin	30 µg	S	R
Chloramphenicol	30 µg	S	S
Ciprofloxacin	5 µg	S	S
Nystatin	100 units	R	
Colistin	25 µg		S

Table S2. Relative abundance of the most dominant bacterial genera in caecal samples of 25-week-old broiler breeders in different flocks (n = 5).

	**Broiler breeder flock**				
**Genus**	A	B	C	SEM	p-value*
Bacteroides	17.41	17.21	17.44	2.62	0.966
Lactobacillus	14.4	17.64	12.00	2.51	0.765
Unknown *Lachnospiraceae*	12.53	12.81	14.94	1.93	0.753
Streptococcus	7.53	12.96	1.94	2.90	0.236
Rikenellaceae RC9 gut group	6.00	2.96	2.12	0.73	*0.071*
Blautia	5.02	5.20	5.43	0.35	0.954
Subdoligranulum	4.14	8.30	7.52	1.34	0.212
Unknown *Bacteroidales*	3.07^{b}	4.19^{b}	0.44^{a}	0.86	**0.035**
Lachnoclostridium	2.78	1.43	2.90	0.33	*0.072*
Romboutsia	2.58	0.92	1.82	0.39	0.207
Ruminococcaceae UCG-014	2.05^{b}	0.51^{a}	$1.01^{a,b}$	0.22	**0.014**
Ruminococcaceae UCG-015	1.99^{b}	0.71^{a}	1.87^{b}	0.23	**0.036**
Turicibacter	1.95^{b}	0.16^{a}	$0.82^{a,b}$	0.34	**0.032**
Christensenellaceae R-7 group	$1.88^{a,b}$	0.75^{a}	2.29^{b}	0.28	**0.023**
Olsenella	1.61	1.36	2.68	0.63	0.147
Alistipes	1.52	0.65	5.26	1.24	0.263
Enorma	1.30	2.08	2.85	0.49	0.183
Collinsella	0.87	1.09	2.99	0.51	0.41
Parabacteroides	0.67	0.29	2.26	0.44	0.467
Fusicatenibacter	0.54	0.72	3.32	0.59	*0.08*
Ruminococcaceae UCG-008	0.32	0.13	1.08	0.16	0.252

Fournierella	0.24	0.09	1.45	0.3	*0.088*
Faecalibacterium	0.24	1.33	0.54	0.31	0.24
Bifidobacterium	0.15	1.21	1.58	0.49	0.925

* = Kruskal-Wallis Test (significant differences are marked in bold, trends in italics)

[a,b] = different letters indicate significant differences within a row (Mann-Whitney Test)

Table S3. Relative abundance of the most dominant bacterial genera in caecal samples of 50-week-old broiler breeders in different flocks (n = 5).

	**Broiler breeder flock**				
Genus	A	B	C	SEM	p-value*
Unknown *Lachnospiraceae*	23.65	23.25	24.85	1.128	0.979
Bacteroides	18.9[b]	12.14[b]	1.58[a]	2.553	**0.012**
Lactobacillus	8.58	8.12	1.33	2.395	0.147
Subdoligranulum	4.97	4.18	7.08	0.497	0.17
Blautia	4.61[a,b]	6.34[b]	1.91[a]	0.58	**0.02**
Akkermansia	3.75	4.42	4.24	1.746	0.888
Lachnoclostridium	3.07	2.83	1.94	0.21	0.179
Romboutsia	2.64	1.54	1.47	0.431	0.463
Turicibacter	2.59	0.69	0.72	0.479	*0.052*
Unknown *Bacteroidales*	2.37	2.2	0.73	0.371	*0.082*
Fusicatenibacter	2.23	1.87	3.71	0.289	0.177
Ruminococcaceae UCG-005	2	2.38	2.75	0.321	0.579
Christensenellaceae R-7 group	1.74[a]	1.44[a]	7.03[b]	0.731	**0.027**
Streptococcus	1.4	1.66	4.11	0.656	0.619
Megamonas	1.35	7.7	0.05	2.443	0.158
Rikenellaceae RC9 gut group	1.22	0.77	1.36	0.234	0.351
Olsenella	1.2	3.91	2.43	0.524	*0.074*
Marvinbryantia	1.14	0.87	1.3	0.149	0.437
Prevotellaceae Ga6A1 group	1.03	0.44	0.13	0.246	0.36
Flavonifractor	0.87	0.46	1.71	0.197	0.36
Sellimonas	0.82[a,b]	1.42[b]	0.29[a]	0.126	**0.001**
Enorma	0.8	2.67	2.02	0.422	0.114
Faecalibacterium	0.63	2.08	1.39	0.284	0.168
Collinsella	0.56	n.d.	2.48	0.525	**0.05**
Parabacteroides	0.45	0.55	0.25	0.053	*0.07*
Bifidobacterium	0.41	8.09	1.12	1.603	*0.053*
Fournierella	0.34	0.13	1.35	0.367	0.585
Alistipes	0.23[b]	2.06[b]	0.06[a]	0.445	**0.044**

Unknown *Eggerthellaceae*	0.22	0.15[a]	1.13[b]	0.113	**0.035**
Tyzzerella	0.21	0.54	3.12	0.452	*0.063*

* = Kruskal-Wallis Test (significant differences are marked in bold, trends in italics)

[a,b] = different letters indicate significant differences within a row (Mann-Whitney Test)

n.d. = not detected

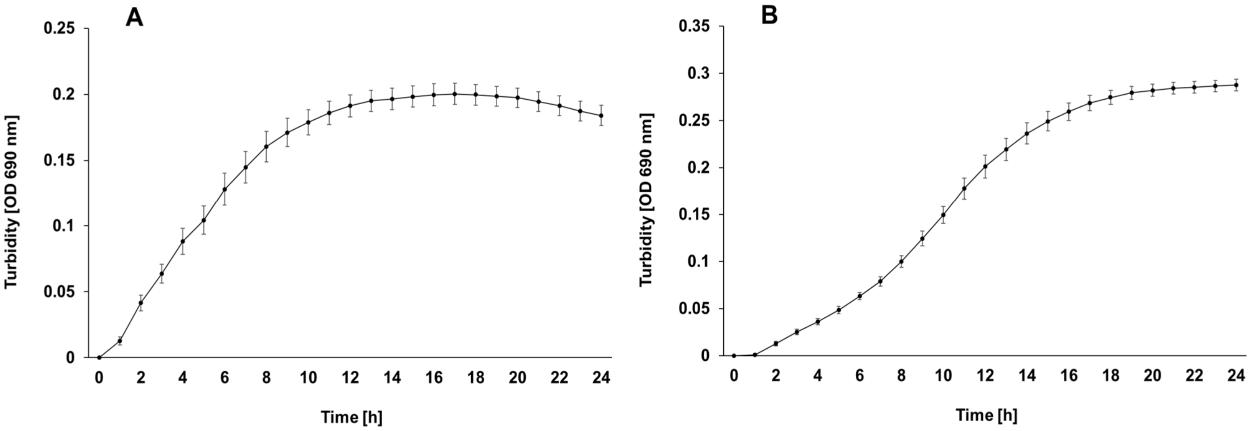

Figure S1. Mean control growth curves of the pathogenic strains *Escherichia coli* O1/O18 (A) and *Salmonella* Enteritidis (B) after incubation in chicken caecal slurries (n = 30), error bars indicate the SEM.
